# Influences of the criminal justice system on use of medications for opioid use disorder: a qualitative study

**DOI:** 10.1186/s44263-024-00093-y

**Published:** 2024-09-19

**Authors:** Emmeline Taylor, Caroline Gray, Matthew Stimmel, Ingrid A. Binswanger, Erica Morse, Christine Timko, Alex H. S. Harris, David Smelson, Andrea K. Finlay

**Affiliations:** 1 Center for Innovation to Implementation (Ci2i), VA Palo Alto Healthcare System, 795 Willow Road (MPD-152), Menlo Park, CA 94025 USA; 2 Department of Clinical Psychology, University of Colorado, Colorado Springs, CO USA; 3 Veterans Justice Programs, U.S. Department of Veterans Affairs, Washington, D.C., USA; 4 Institute for Health Research, Kaiser Permanente Colorado, Aurora, CO USA; 5 Colorado Permanente Medical Group, Denver, CO USA; 6 Division of General Internal Medicine, University of Colorado School of Medicine, Aurora, CO USA; 7 Department of Health Systems Science, Bernard J Tyson Kaiser Permanente School of Medicine, Pasadena, CA USA; 8 Department of Psychiatry and Behavioral Sciences, Stanford University School of Medicine, Palo Alto, CA USA; 9 Department of Surgery, Stanford University School of Medicine, Palo Alto, CA USA; 10 Center for Organization and Implementation Science, Edith Nourse Rogers VA Medical Center, Bedford, MA USA; 11 Department of Veterans Affairs, National Center On Homelessness Among Veterans, Washington, D.C., USA; 12 Schar School of Policy and Government, George Mason University, Arlington, VA USA

**Keywords:** Veteran, Criminal Justice, Opioid Use Disorder, Qualitative Research

## Abstract

**Background:**

Legal-involved veterans with opioid use disorder (OUD) have lower receipt of medications for opioid use disorder (MOUD) than other veterans served at the Veterans Health Administration (VHA). This qualitative study examined the influence of the criminal justice system on access to MOUD for legal-involved veterans in the U.S.

**Methods:**

VHA facilities (*n* = 14) that varied in their provision of MOUD to legal-involved veterans were selected for qualitative interviews. Interviewees included legal-involved veterans (*n* = 18), VHA Veterans Justice Programs Specialists (*n* = 15), substance use disorder treatment providers (*n* = 5), and criminal justice staff (*n* = 12). Team members applied codes to meaningful units of analysis (quotations) in the transcribed interviews. Using a matrix approach, team members created a spreadsheet matrix with codes, facility rate of MOUD, and relevant quotations summarized for each participant. Themes and connections between individual participants and cross-interview concepts were explored. Participants were not asked to provide feedback on the findings.

**Results:**

Themes identified were as follows: (1) Veterans Treatment Court policies both enhanced and limited MOUD treatment access and utilization among participants; (2) cross-system collaboration strengths and challenges existed; and (3) criminal justice system treatment preferences and policies both enhanced and limited MOUD in jails and prisons.

**Conclusions:**

The influence of the criminal justice system on MOUD has led to variable access to MOUD for legal-involved veterans. Our findings can help inform recommendations to enhance access to MOUD for veterans within the criminal justice system, including the development of a national database of MOUD education materials for Veterans Treatment Courts, strengthening community-court relationships, allowing individuals to use their own healthcare coverage within jails and prisons and extend Medicaid coverage into criminal justice settings, and applying national quality measures for MOUD to criminal justice settings and develop a national system for tracking these quality measures.

**Supplementary Information:**

The online version contains supplementary material available at 10.1186/s44263-024-00093-y.

## Background

United States (U.S.) military veterans with criminal justice system involvement, compared to those without, have an elevated opioid-related overdose mortality risk [1]. Medications for opioid use disorder (MOUD) effectively treat opioid use disorder (OUD) and reduce overdose risk [2, 3]. Yet, many barriers prevent consistent support, adoption, and use of MOUD across the criminal justice system and collaborating healthcare agencies [4–6]. Previous studies suggest that stigma, lack of education around MOUD, provider shortages, and MOUD treatment “deserts” are barriers to MOUD within communities and healthcare systems [4, 7].

The criminal justice system can also impact veterans’ ability to access MOUD [4, 8]. Criminal justice system barriers to MOUD have historically included policies restricting MOUD to specific sub-groups (e.g., pregnant women) and abstinence-oriented environments [5]. Some jails and prisons do not offer or maintain MOUD in their facilities, further limiting MOUD options [8]. For veterans, the Veterans Health Administration (VHA) cannot provide or pay for healthcare for incarcerated veterans. Historically, Medicaid could not be used to pay for healthcare, though recent policy changes allow states to request Medicaid pre-release services up to 90 days prior to release from prison or jail [9, 10]. The Stanford-*Lancet* Commission recommended offering universal substance use care, including MOUD in criminal justice settings, tailored to individual’s needs. They also recommended that incarcerated individuals have access to MOUD, including after release from jail or prison, when the risk of overdose is greatest [11].

The VHA is the largest integrated healthcare system in the U.S., providing medical and mental healthcare to approximately 9 million veterans each year. The VHA’s Veterans Justice Programs (VJP) provides outreach to veterans in criminal legal settings, including law enforcement, courts, jails, and prisons [12]. Criminal legal agencies include Veterans Treatment Courts, which are based in the county court system and are a veteran-specific hybrid of drug and mental health courts [13]. Veterans Treatment Courts are similar to other problem-solving courts but also include unique attributes, such as veteran mentors and resources and treatment available through the VHA system. Staff from VHA, known as Veterans Justice Programs Specialists, work with their partners in criminal justice agencies to assist veterans. However, all criminal legal agencies, including Veterans Treatment Courts, are separate entities from VHA. Services offered to veterans in criminal legal agencies are at the discretion of those agencies. VJP Specialists identify legal-involved veterans in criminal legal settings, assess their treatment needs, and facilitate access to VHA services, including substance use treatment and MOUD [12, 14].

Within the VHA, mandates exist to consider MOUD for all indicated veterans [15]. However, legal-involved veterans still use MOUD less than non-legal-involved veterans [16]. Barriers to MOUD identified for legal-involved veterans mirror barriers to MOUD for other populations and include stigma towards medications and concerns by the justice systems about non-prescription use and diversion [4].

This study aimed to qualitatively examine the influence of the criminal justice system on access to MOUD for legal-involved veterans from the perspectives of staff who work within these systems and veterans themselves. Courts, jails, and prisons are three entities that can offer or connect veterans with MOUD. As the VHA continues to expand its collaborations with non-VHA entities, identifying and addressing barriers created by the interaction of these disparate systems, as well as understanding the mechanisms for success in communities where these systems are working well together, will ensure more legal-involved veterans have access to these life-saving medications.

## Methods

### Study design

The present qualitative study follows the Standards for Reporting Qualitative Research (SRQR) guidelines (see Additional file 1) [17]. This study developed from a larger mixed-methods project which quantitatively examined receipt of MOUD among legal-involved veterans who received treatment at VHA facilities and qualitatively examined experiences around access to and availability of MOUD for legal-involved veterans [4, 16].

### Researcher characteristics and reflexivity

This study was conducted by a multidisciplinary team. Members of the team involved in interviews and analysis included a doctoral-level research health scientist (AF), a physician (IB), a medical anthropologist (EM), and three research assistants with bachelor’s degrees in psychology (ET, RK, LM). Four members worked in the VHA, and two in health system and academic medicine settings. All members identified as women, and their racial and ethnic identity included White, African-American, and Asian. The Principal Investigator (AK), a Co-Investigator (IB), and Project Manager (EM) all had extensive background in legal-involved populations and mixed-methods and qualitative study designs. The Principal Investigator and Project Manager led training and overseeing research assistants in qualitative methods. The researchers did not have relationships with participants apart from study interactions.

### VHA facility selection

To guide VHA facility selection for inclusion in the study, quantitative electronic health record data were used to rank VHA facilities nationally based on MOUD receipt rates among legal-involved and non-legal-involved veterans. VHA facilities were ranked by high or low performance, that is, high-performing facilities had a higher proportion of legal-involved veterans who had received MOUD compared to non-legal-involved veterans, whereas low-performing facilities had a lower proportion in fiscal year 2017. Facilities were also ranked by change in MOUD receipt. Increasing and decreasing facilities were defined as facilities where a larger and smaller proportion of legal-involved veterans, respectively, received MOUD in Fiscal Year 2017 compared to Fiscal Year 2016. Fourteen facilities representing high, increasing, low, and decreasing rates of MOUD receipt were selected for qualitative interviews.

### Sample

Four groups of participants within or that interacted with selected VHA facilities were recruited to complete interviews: (1) legal-involved veterans served by selected facilities, (2) VHA VJP Specialists, (3) substance use disorder treatment providers within the VHA facilities or serving facilities but in the local community, including psychiatrists and therapists specializing in the treatment of substance use disorders, and (4) local criminal justice system staff, including judges, court staff, and probation officers.

Participant eligibility requirements were age 18 years or older, English speaking, and able to understand study procedures. Legal-involved veterans were included if they had a history of opioid use or opioid use disorder within the last 10 years and a history of criminal legal involvement, defined as having been arrested, in jail or prison, on probation or parole, or in criminal court within the last 10 years, but not incarcerated at the time of the interview. VJP Specialists, substance use disorder treatment providers, and criminal justice staff were eligible if they served in that role at the time of the interview.

#### Sample characteristics

Participants were recruited from 14 selected VHA facilities located in the Northeast (*n* = 6), South (*n* = 9), Midwest (*n *= 12), and West (*n* = 23). Across the 14 facilities, 50 participants completed interviews: 18 legal-involved veterans, 15 VJP Specialists, 5 treatment providers (4 from VHA, 1 from the community), and 12 criminal justice staff. Treatment providers included 2 medical doctors who could prescribe MOUD and 3 masters or doctorate-level providers who provided behavioral treatment. Criminal justice staff included 2 judges, 8 court staff, and 2 probation officers. Thirty-two percent of participants were connected with facilities with decreasing rates of MOUD, followed by 26% connected to facilities with high rates of MOUD, 15% connected to facilities with low rates of MOUD, and 10% connected to facilities with increasing rates of MOUD.

### Procedures

Study staff directly contacted VJP Specialists via email to invite them to participate in semi-structured interviews (see Additional file 2 for interview guide). VJP Specialists who agreed to participate were asked to provide study recruitment flyers to potential participants, including substance use disorder treatment providers, criminal justice staff, and veterans who met eligibility requirements. VJP Specialists also asked potential participants if they could share their contact information with the research team. Recruitment and interviewing occurred from February 2018 through March 2019.

After reviewing informed consent, interviews were conducted by telephone or in-person and ranged between 15 and 60 min. All interviews were audio-recorded, deidentified, and transcribed verbatim. Transcripts were not returned to participants for comment or correction. A $30 incentive was offered to legal-involved veterans and non-VHA employed participants (criminal justice staff and community substance use disorder treatment providers) for their participation; most criminal justice staff declined because of court or grant policies prohibiting receipt of monetary incentives. Due to VHA restrictions on research incentives, VHA staff were not offered incentives to participate.

### Interview topics

Participants were asked questions about barriers and facilitators to accessing MOUD, knowledge of and preferences for different MOUD, philosophy towards addiction treatment, and how the criminal justice system affected OUD treatment. Legal-involved veterans were asked about their legal system involvement and experiences with the criminal justice system. This study focused on participants’ perspectives of the influence of the criminal justice system on access to MOUD among legal-involved veterans, and analyses were limited to questions and responses relevant to this focus.

### Analysis

Interview transcripts were analyzed in ATLAS.ti (ATLAS.ti; Version 8, Berlin, Germany) [19]. The Principal Investigator, a Co-Investigator, the project manager, and three research assistants conducted analyses. The entire team met regularly throughout the interview process to discuss whether interviews yielded enough information to answer the research questions and reach thematic saturation. A total of 50 interviews were conducted which is more than the minimum number of interviews others have suggested are necessary for reaching saturation, particularly for studies that focus on addressing a more delimited set of research questions [20, 21].

The research team took a deductive and inductive approach to coding. An a priori code list was created from constructs informing the interview questions. The code list was then revised throughout the interview process to include additional codes which mapped onto emerging themes within the data from the interviews. Code groups were created to combine similar codes to assist with data organization and the development of the analytical framework (see Additional file 3 for list of codes). Team members applied codes to meaningful units of analysis (quotations) in the transcribed interviews. Team members used a matrix approach to create a spreadsheet matrix with codes, facility rate of MOUD (i.e., high, low, increasing, decreasing), and relevant quotations summarized for each participant [22]. The matrix approach was used to discuss themes and explore connections between individual participants and cross-interview concepts. Participants were not asked to provide feedback on the findings.

## Results

### Identified themes

Themes were limited to the influence of the criminal justice system on MOUD for legal-involved veterans. Three themes were identified: (1) Veterans Treatment Court policies both enhanced and limited MOUD treatment access and utilization among participants; (2) cross-system collaboration strengths and challenges existed; and (3) criminal justice system treatment preferences and policies both enhanced and limited MOUD in jails and prisons. Taken together, the themes highlighted varying perspectives on the criminal justice system’s influence on MOUD for legal-involved veterans, suggesting that MOUD access and cross-system collaboration may be more successful in some communities compared to others.

### Veterans treatment court policies both enhanced and limited MOUD treatment access and utilization among participants

Broadly across all participant groups, perspectives were varied around whether court mandates limited or facilitated MOUD access within these settings, suggesting inconsistency in how MOUD is viewed and used within the VHA population involved with Veterans Treatment Courts. Among participants connected with VHA facilities that were either increasing or high in their rates of MOUD, participants reported that courts listened to medical provider recommendations and supported MOUD by not limiting it in their policies, thus allowing for more successful treatment and better access to MOUD. For example, a probation officer associated with an increasing facility highlighted the importance of differentiating between healthcare and legal issues and how choices around MOUD should be left in the hands of medical providers. He explained,

As far as medically-assisted treatment, as an officer of the Court, if the doctor writes a prescription for something, we don’t touch that. We don’t engage ourselves in any kind of medical care. We’re the only state that sees that as the right of the healthcare rather than a legal issue. (ID 116, Probation Officer, Facility with an Increasing Rate of MOUD)

Many veterans at facilities with high rates of MOUD appreciated the willingness of judges to listen to the recommendations of clinicians who encouraged access to MOUD while in treatment court, although this varied by region with less support for MOUD at facilities in the Northeast with decreasing rates of MOUD. VJP Specialists and providers associated with facilities with high MOUD rates noted that they had seen changes within the Veterans Treatment Courts around openness to MOUD.

Things have gotten better and the courts are getting more used to [MOUD] in general and [it is] becoming way more accepted. When they have more experience with it, the better it is. (ID 133, VJP, Facility with a High Rate of MOUD)

Some participants highlighted rules barring MOUD within Veterans Treatment Courts. This appeared to be more of a challenge within courts connected to facilities with low and decreasing rates of MOUD. Veterans who had participated in Veterans Treatment Courts connected to VHAs across rates of MOUD highlighted concerns about court mandates and policies requiring OUD treatment and specific types of treatment or medications and how that affected MOUD access and program completion. For example, a veteran shared that they could not graduate from Veterans Treatment Court without stopping MOUD treatment:

It takes longer to I guess get through the program because you have to have so many days clean, and that’s off [MOUD] before you can graduate [from Veterans Treatment Court]. (ID 135, Vet, Facility with a Decreasing Rate of MOUD)

Criminal justice staff associated with VHA facilities with varying rates of MOUD described court mandates for specific types of treatment as an integral part of Veterans Treatment Courts, and in some instances, conflated participation in the Veterans Treatment Court with treatment itself.

The VA substance abuse programs are more lenient and there are not enough long-term residential programs for veterans who need them. Whereas our treatment programs are more stringent with respect to attendance, supervised drug testing, random drug testing, things that we require in treatment court. (ID 123, Judge, Facility with a Low Rate of MOUD)

VJP Specialists mentioned court policies barring specific MOUDs and requiring other types of treatment, such as residential treatment. One VJP Specialist explained:

[Despite veteran treatment preference], the court is most likely mandating residential because of their public status or [the veteran’s] need to be under closer supervision, so I’d let the court know what their preference is, but it’s not their decision. (ID 103, VJP, Facility with a Low Rate of MOUD)

VJP Specialists at increasing and high facilities shared that preferences for, and education around, MOUDs enhanced access to MOUD in some criminal justice entities. However, some criminal justice organizations and staff, more commonly at low and decreasing facilities, abided by abstinence-only philosophies and preferred psychotherapy-only treatments, which hindered access to MOUD. Some veterans across all facility rates of MOUD shared their perception that the criminal justice system preferred psychotherapy to be the primary treatment for OUD. For example:

From what I’ve seen and what I’ve experienced [MOUD] is an option, but it’s not necessarily their first option. It is a weapon in their arsenal […], but it’s not their main one. […] I believe it’s therapy first. (ID 117, Vet, Facility with a High Rate of MOUD)

VJP Specialists identified the broader justice system’s culture as a factor limiting access to MOUD. For example:

[…] it’s the personality of the judge or the magistrate that determines whether they’re going to give some latitude to a veteran or not. I go to the statewide conference […] primarily for judges and court personnel, and many of them just believe that Suboxone, methadone, and other treatments like that was just swapping one addiction for another. (ID 113, VJP, Facility with a High Rate of MOUD)

Substance use disorder treatment providers at facilities with low rates of MOUD perceived court mandates for behavioral treatment as sometimes leading to challenges with engagement among veterans. Providers felt this was especially true when veterans viewed the motivation for reducing their substance use as external rather than internal. The veterans they worked with often identified feeling forced to participate in inpatient or outpatient substance use disorder treatment because of court mandates. Providers and VJP Specialists at decreasing and low facilities mentioned that courts can recommend or mandate treatment, which can be at odds with the medical provider’s recommendation. Additionally, it was highlighted that some courts overlook the opinions of VHA medical providers who have specialized training in understanding the health needs of veterans. For example, one VJP Specialist pointed out:

There’s a lot of obstruction[...]. [The court] will have community mental health providers sit in at the Vet Court staffing even though they aren’t part of the Vet Court team. The judge will ask their opinions on [treatment] issues, on veterans that they’ve never seen before. (ID 107, VJP, Facility with a Low Rate of MOUD)

A few VJP Specialists at decreasing facilities discussed how criminal justice system entities would outright ignore and not follow medical provider recommendations. One stated:

One of [my] vet court guys was on Suboxone. Well, we needed to call the judge because, ultimately if you’re in vet court, it’s the judge’s decision even though that should not be the case because he is not a doctor. […] so we called the judge, and he said no. Even though it’s really not supposed to be like that because [the judge] is supposed to support the [medical] provider. (ID 105, VJP, Facility with a Decreasing Rate of MOUD).

#### Cross-system collaboration strengths and challenges existed

There were veterans, VJP Specialists, providers, and criminal justice staff who reported strong collaboration across systems when meeting veterans’ treatment needs, and this was consistent across facility MOUD rates. VJP Specialists and criminal justice staff noted effective partnership with community providers around getting care for VA- and non-VA-connected veterans. Additionally, some VJP Specialists recognized their criminal justice system collaborators for their attention towards the unique needs and issues faced by legal-involved veterans. For example:

We work really well together and collaborating and they understand the various issues that justice-involved veterans are facing besides addiction, so you know, the myriad of issues and we work pretty well together. (ID 103, VJP, Facility with a Low Rate of MOUD).

Providers at high, low, and decreasing facilities also identified positive collaboration with jails and prisons around coordinating access to veterans’ medical records and medications in jail/prison or before release to ensure continuity of care and access to MOUD. Criminal justice staff members described successful relationships with VHA VJP Specialists and substance use providers in coordinating treatment for veterans. Similarly, veterans highlighted effective coordination between the VHA and Veterans Treatment Courts leading to access to MOUD.

Nevertheless, challenges were also mentioned. VJP Specialists across all facility rates of MOUD pointed out challenges in coordinating MOUD access for veterans following their release from jail or prison due to inconsistent communication from the jails and prisons around a veteran’s release date. Stigma around veterans’ legal involvement was mentioned by VJP Specialists as limiting collaboration with residential treatment programs that were apprehensive about accepting legal-involved veterans. They also highlighted how stigma towards MOUD impacted some judges’ willingness to collaborate with VJP Specialists and Providers on MOUD treatment access for legal-involved veterans.

A few veterans across all facility rates of MOUD mentioned a lack of communication between systems which affected their experience in treatment court. For example:

They don’t communicate at all. They just don’t. In fact, there were times [in court] when I was getting in trouble for something, and my counselor here at the VA wrote letters on my behalf, and they weren’t even considered. (ID 134, Vet, Facility with a High Rate of MOUD)

Criminal justice staff connected to facilities with decreasing and high rates of MOUD also shared how a lack of VJP Specialists in some geographic areas negatively impacted care coordination, including MOUD access, for legal-involved veterans. For example, a staff person working in the Veterans Trauma Court shared:

I think we had over 450 veterans in the jail last year, and I think that would justify a full position to be in the jail every day. I think [our VJP Specialist] goes in once a week, if that. […] I think it would be an additional [VJP Specialist] that we would benefit from. (ID 132, Court Staff, Facility with a High Rate of MOUD).

#### Criminal justice system treatment preferences and policies both enhanced and limited MOUD in jails and prisons

Veterans, VJP Specialists, and criminal justice staff reported that in some areas, the criminal justice system was supportive of MOUD in jails and prisons. Nevertheless, across facility rates of MOUD, Veterans and VJP Specialists expressed frustration around the justice system determining MOUD use and the type of available MOUD. One veteran stated:

The problem [around access] doesn’t lie within the patient and the VA. I think it lies with the actual justice system; they are the ones that make these decisions and they’re the ones that decide whether you can or cannot [use medications]. (ID 117, Vet, Facility with a High Rate of MOUD)

Veterans, VJP Specialists, and providers across facilities rates of MOUD highlighted how the lack of availability and access to MOUD in jail or prison affected veterans’ medical care. One veteran described the detoxification experience during incarceration:

It was awful. It was worse than dogs in a kennel. They literally laughed at me, threw me in a closet basically, and let you sweat it out. (ID 117, Vet, Facility with a High Rate of MOUD)

Overall, the lack of access to MOUD in jails and prisons was perceived as forcing some veterans to end their MOUD treatment, increasing their risk for overdose following release and leading to additional challenges in re-accessing MOUD. Providers highlighted this danger:

Detoxing people while they’re incarcerated and having them go back out into the community immediately puts them at risk for dying […]. Which is why it’s probably better to put them on evidence-based medication-assisted therapy, and then continue them on it when they go out, to reduce risk of overdose. (ID 137, VHA Substance Use Provider, Facility with a Decreasing Rate of MOUD)

Providers at decreasing facilities mentioned that only certain medications were allowed in some jails and prisons, which meant that a veteran had to discontinue one type of MOUD to be placed on another that was available and approved by the prison. Similarly, veterans shared that access to MOUD in jails and prisons was inconsistent. For example:

There’s been a couple of times where I had been on methadone, and I went to jail, and [...] they don’t support giving methadone or Suboxone over there. It really is a hard experience [...] to withdraw from methadone because you can’t get your medication while you’re in jail. (ID 153, Vet, Facility with a Decreasing Rate of MOUD)

Across all facility rates of MOUD, providers and criminal justice staff also shared ways in which access to MOUD within the criminal justice system more broadly (i.e., jails, prisons, and courts) had been constrained by systemic and structural issues. Specifically, insurance challenges, lack of VA benefit eligibility, cost, and lack of MOUD prescribing providers were all cited as barriers affecting MOUD access for legal-involved veterans. Additionally, delays connecting veterans released from incarceration to outpatient MOUD treatment were a barrier. Providers highlighted a history of policies that have limited access to MOUD and felt with recent policy changes promoting MOUD within correctional settings, MOUD would likely become more accessible. However, they did not provide specific policy language or documentation.

## Discussion

This study identified three themes related to the influence of the criminal justice system on MOUD for legal-involved veterans in the U.S.: (1) Veterans Treatment Court policies both enhanced and limited MOUD treatment access and utilization among participants; (2) cross-system collaboration strengths and challenges existed; and (3) criminal justice system treatment preferences and policies both enhanced and limited MOUD in jails and prisons. Broadly, the themes suggest that the criminal justice system plays a vital role in legal-involved veterans’ access to care for OUD, specifically MOUD, and that their role as facilitating or limiting is variable based on region and facility rates of MOUD. Generally, participants associated with VHA facilities with low and decreasing rates of MOUD reported policies barring MOUD within jails, prisons, and Veteran Treatment Courts. Given that these entities were less supportive of MOUD, it may be a factor contributing to the decreasing or low rates of MOUD at the connected VHA facilities.

Best practice standards for treatment courts in the U.S. highlight the need to (1) universally offer MOUD, and (2) provide access to all available medications [23]. Similar recommendations exist for jails and prisons, yet universal access to MOUD in U.S. legal settings has not occurred [18, 24]. As of 2021, only nine states required their state prisons to provide access to all U.S. Food and Drug Administration (FDA)-approved MOUDs [25]. State policy reform has occurred following a series of court decisions mandating MOUD access within jails and prisons on the grounds of the Americans with Disabilities Act and the U.S. Constitutional Eighth Amendment prohibiting cruel and unusual punishment [26]. Although state policies are emerging that require access to MOUD in correctional settings and a failure to do so is liable to be litigated, many of the policies do not explicitly include Veterans Treatment Courts, nor are they being universally adopted.

Since study data collection, a U.S. federal ruling allowing buprenorphine treatment via telehealth was established during and following the COVID-19 pandemic, yet a study of the implementation of telemedicine MOUD in community health centers in 2021 found inequities in access because of challenges in access to telemedicine technology [27]. In a qualitative study examining telemedicine in jails and prisons during the COVID-19 pandemic, reduced availability of community-based health care to provide post-release telehealth support emerged as a barrier to MOUD access via telehealth [28]. Additionally, despite the increase in telemedicine for MOUD, as of 2022, only about 32% of jails initiated MOUD when indicated [29]. Consistency in adhering to mandates to provide MOUD has been variable, and uptake of new policies designed to increase MOUD access is not always aligned with best practice standards. The present study highlights variability in MOUD access, and based on the themes identified in this study, we provide recommendations for enhancing access to MOUD and ensuring uniformity in that access for legal-involved veterans within the criminal justice system.

Recommendation 1: Develop a national database of MOUD education materials for Veterans Treatment Courts. VJP Specialists at increasing and high facilities shared that MOUD access seemed to be enhanced when criminal justice entities were educated on types of MOUDs and their use as a gold-standard for the treatment of OUD. Additionally, participants highlighted abstinence-only beliefs as interfering with MOUD uptake and use. Educating systems on the evidence supporting the use of MOUD with justice-involved populations may be a critical way to enhance MOUD access. Consistent with the present study, a 2023 qualitative study with social service clinicians from a state department of corrections found naltrexone initiation was enhanced related to positive judge and probation officer attitudes around naltrexone, whereas buprenorphine stigma by criminal justice staff was a barrier to treatment [30].

The U.S.-based National Drug Court Institute developed a toolkit to support treatment courts in implementing systems to make MOUD available [23, 31]. The toolkit highlights the current evidence supporting MOUD as a gold-standard for treatment, as well common principles and best practices that have supported MOUD access in other courts. Ensuring that all Veterans Treatment Courts receive education and training on this toolkit and support in implementation may help facilitate increased access to MOUD for veterans by reducing stigma and increasing knowledge around the scientific evidence supporting MOUD. Additionally, augmenting the toolkit to include more specific information on VHA and community resources available for legal-involved veterans trying to access MOUD may support connection to these medications, as well as cross-system collaboration for the veteran population.

Recommendation 2: Strengthen community-court relationships. A strength consistently mentioned by study participants across facility rates of MOUD was effective communication and collaboration between key entities working with the Veterans Treatment Courts. It seems likely that these relationships can be used to facilitate increased uptake of MOUD in these settings. The results suggest that courts associated with facilities with high and increasing rates of MOUD, see the value in continued education on MOUD and enhancing MOUD. At low and decreasing facilities, using the strong collaborative spirit highlighted by interviewees could be a way to then increase education for the justice system through VJP Specialists and VHA providers who have specific training in MOUD and the evidence for MOUD as an effective treatment for OUD.

Given that the present study highlighted how strong collaboration helped to facilitate access to MOUD, encouraging organizations, like VHA, and courts to formalize and strengthen relationships may allow for the pooling of resources, education, and knowledge that would likely support MOUD access. For example, VHA has established data-sharing agreements and medical-legal partnerships with various treatment courts and legal entities [32]. Previous research suggests that collaboration across systems relates to treatment courts’ positive beliefs in the trustworthiness and efficacy of MOUD, further highlighting the importance of developing these relationships [33]. Research conducted in Massachusetts jails indicated that mandates alone were not enough for successful implementation of MOUD — collaboration and reliance on external agencies were necessary components [34]. Similarly, a 2023 pilot study examined the initiation of extended-release naltrexone 30 days prior to jail release by a community health organization and local criminal justice entities [35]. In their assessment of feasibility, researchers found that the establishment of a strong partnership between the criminal justice agencies and health organizations was essential to the success of the implementation.

Recommendation 3: Allow individuals to use their own healthcare coverage within jails and prisons and support states in requesting extensions of Medicaid coverage into criminal justice settings. In interviews, criminal justice system policies were cited as limiting access to MOUD for legal-involved veterans. Specifically, insurance challenges, lack of VHA eligibility, cost, and lack of providers were all cited as barriers affecting MOUD access for legal-involved veterans. Prior research aligns with the perspectives in this study and highlights barriers such as cost for the jails and prisons, lack of independent healthcare services within jails and prisons, and high co-payments for legal-involved individuals as limiting access to quality healthcare, including MOUD [36, 37]. Local policies against MOUD, differences in criteria used to establish MOUD eligibility, lack of resources within criminal justice settings, and differing opinions around MOUD diversion risk can impact access [38–40]. Although organizations, including the National Sheriffs’ Association, the American Society of Addiction Medicine, and the Substance Abuse and Mental Health Services Administration, promote access to MOUD in criminal justice settings, inconsistency in care options within these settings, as described in this study, and a lack of federal standards help maintain variable access to MOUD [41–44]. Barriers to providing individuals with quality healthcare, including MOUD, represent liability issues for jails and prisons. Yet, many of these settings lack the resources to provide quality care.

In 2022, the Council on Criminal Justice proposed moving towards policy changes that would allow for expanded Medicaid access in these settings to better meet the health needs of legal-involved individuals who often qualify for Medicaid, thus reducing resource burden on jails and prisons [45]. In 2023, the U.S. Department of Health and Human Services, Centers for Medicare and Medicaid Services announced an opportunity for state Medicaid to cover substance use-related services for up to 90 days prior to the individual’s expected release date that could not otherwise be covered by Medicaid due to previous policies prohibiting Medicaid payment for most services provided to most incarcerated people [10]. Some legal-involved veterans qualify for healthcare through VHA, which similar to Medicare and Medicaid, is a federal health insurance source. An area for future research could examine how developing a program that would allow these individuals to access MOUD and other healthcare resources from VHA during incarceration might improve access to MOUD for legal-involved veterans.

Recommendation 4: Apply national quality measures for MOUD to criminal justice settings and develop a national system tracking these quality measures. Across all themes in the present study, there was notable variation in MOUD access, as well as barriers and facilitators to MOUD access. Consistent access to MOUD across systems and geographic areas is necessary for ensuring justice-involved veterans have the opportunity to use this evidence-based treatment for OUD. Implementing national data collection around medical care and health provides an opportunity for systems to collaborate on ensuring healthcare quality and access to care, including MOUD [43, 46]. Having clear quality measures identified and tracked may allow for the VHA, community providers, and the criminal justice system to better collaborate on access to MOUD for legal-involved veterans and to provide more consistent access. Additionally, a national tracking system will allow for identification of Veterans Treatment Courts and other criminal justice settings that need additional support and resources to provide high-quality MOUD care.

### Limitations

The present study is not without limitations. Few providers responded to recruitment requests, and providers were not asked if they were certified in addiction medicine. Additionally, we did not ask how much authority criminal justice staff had in treatment decisions for veterans. Participants were not randomly selected for interviews, and therefore we may have missed other themes that would have been identified in a random sample. Additionally, the findings may lack generalizability to other non-veteran legal-involved populations. Legal-involved veterans were required to have legal involvement within 10 years before study recruitment, and thus some of the perspectives presented may not be related to the current VHA and justice system environment. Recommendations and policies for access to MOUD in correctional settings were issued in 2015 and 2020 [43]. Some perspectives shared in this study may be related to experiences before formal recognition of the need for MOUD within these settings, and interviews for this study were conducted between February of 2018 and March of 2019, prior to the release of the 2020 recommendations and policies. Nevertheless, the implementation and adherence to these policies are far from universal [43].

## Conclusions

This study identified three themes on the influence of the criminal justice system on MOUD for legal-involved veterans: (1) Veterans Treatment Court policies both enhanced and limited MOUD treatment access and utilization among participants; (2) cross-system collaboration strengths and challenges existed; and (3) criminal justice system treatment preferences and policies both enhanced and limited MOUD in jails and prisons. The provided recommendations addressing these themes may enhance access to MOUD for legal-involved veterans within the criminal justice system. Specifically, developing a national database of MOUD education materials for Veterans Treatment Courts, strengthening community-court relationships, allowing individuals to use their own healthcare coverage within jails and prisons and extend Medicaid coverage into criminal justice settings, and applying national quality measures for MOUD to criminal justice settings and develop a national system tracking these quality measures.

## Supplementary Information

Additional file 1: Standards for Qualitative Research (SRQR) Guidelines

Additional file 2: Qualitative Interview Guide

Additional file 3: Analysis Code Book

## Data Availability

The United States Department of Veterans Affairs (VA) places legal restrictions on access to veteran’s health care data, which includes both identifying data and sensitive patient information. The analytic data sets used for this study are not permitted to leave the VA firewall without a Data Use Agreement. For more information, please visit https://www.virec.research.va.gov or contact the VA Information Resource Center (VIReC) at virec@va.gov.

## Abbreviations

FDA: U.S. Food and Drug Administration
MOUD: Medications for opioid use disorder
OUD: Opioid use disorder
SRQR: Standards for Reporting Qualitative Research
U.S.: United States
VHA: Veterans Health Administration
VJP: VHA’s Veterans Justice Programs

## References

[1] Finlay AK, Palframan KM, Stimmel M, McCarthy JF. Legal system involvement and opioid-related overdose mortality in U. S. Department of Veterans Affairs patients. Am J Prev Med. 2022;62(1):e29-37. 34521559 10.1016/j.amepre.2021.06.014 PMC8849578

[2] Babu KM, Brent J, Juurlink DN. Prevention of opioid overdose. N Engl J Med. 2019;380(23):2246–55. 31167053 10.1056/NEJMra1807054

[3] Larochelle MR, Bernson D, Land T, Stopka TJ, Wang N, Xuan Z, et al. Medication for opioid use disorder after nonfatal opioid overdose and association with mortality. Ann Intern Med. 2018;169(3):137–45. 29913516 10.7326/M17-3107 PMC6387681

[4] Finlay AK, Morse E, Stimmel M, Taylor E, Timko C, Harris AHS, et al. Barriers to medications for opioid use disorder among veterans involved in the legal system: a qualitative study. J Gen Intern Med. 2020;35(9):2529–36. 32583337 10.1007/s11606-020-05944-6 PMC7459011

[5] Grella CE, Ostile E, Scott CK, Dennis M, Carnavale J. A Scoping review of barriers and facilitators to implementation of medications for treatment of opioid use disorder within the criminal justice system. Int J Drug Policy. 2020;81:102768. 32446130 10.1016/j.drugpo.2020.102768 PMC8372195

[6] Komalasari R, Wilson S, Haw S. A systematic review of qualitative evidence on barriers to and facilitators of the implementation of opioid agonist treatment (OAT) programmes in prisons. Int J Drug Policy. 2021;87:102978. 33129135 10.1016/j.drugpo.2020.102978

[7] Rosenblatt RA, Andrilla CHA, Catlin M, Larson EH. Geographic and specialty distribution of US physicians trained to treat opioid use disorder. Ann Fam Med. 2015;13(1):23–6. 25583888 10.1370/afm.1735 PMC4291261

[8] Winetsky D, Fox A, Nijhawan A, Rich JD. Treating opioid use disorder and related infectious diseases in the criminal justice system. Infect Dis Clin North Am. 2020;34(3):585–603. 32782103 10.1016/j.idc.2020.06.012 PMC7433020

[9] U.S. Department of Veterans Affairs enrollment provisions and medical benefits package( (§ 17.3838 C.F.R. § 17.38(c)(5)). Washington, D.C.: U.S. Department of Veterans Affairs.

[10] Social Security Act (42 U.S.C. § 1315 Medicaid Reentry Section 1115 Demonstration Opportunity). Washington, D.C.: Department of Health and Human Services.

[11] Humphreys K, Shover CL, Andrews CM, Bohnert ASB, Brandeau ML, Caulkins JP, et al. Responding to the opioid crisis in North America and beyond: recommendations of the Stanford-Lancet Commission. Lancet. 2022;399(10324):555–604. 35122753 10.1016/S0140-6736(21)02252-2 PMC9261968

[12] Blue-Howells JH, Clark SC, van den Berk-Clark C, McGuire JF. The U. S. Department of Veterans Affairs Veterans Justice Programs and the sequential intercept model: case examples in national dissemination of intervention for justice-involved veterans. Psychol Serv. 2013;10(1):48–53. 22924802 10.1037/a0029652

[13] Russell RT. Veterans treatment court: a proactive approach. N Engl J Crim Civ Confin. 2009;35(2):357–72.

[14] VHA Directive Veterans Justice Programs (1162.06.). Washington, D.C., U.S. Department of Veterans Affairs.

[15] Uniform Mental Health Services in VA medical centers and clinics (VHA Handbook 1160.1). Washington, D.C., U.S. Department of Veterans Affairs.

[16] Finlay AK, Harris AHS, Timko C, Yu M, Smelson D, Stimmel M, et al. Disparities in access to medications for opioid use disorder in the Veterans Health Administration. J Addict Med. 2020;15(2):143–49. 10.1097/ADM.0000000000000719 PMC7889740 32826617

[17] O’Brien BC, Harris IB, Beckman TJ, Reed DA, Cook DA. Standards for reporting qualitative research: a synthesis of recommendations. Acad Med J Assoc Am Med Coll. 2014;89(9):1245–51. 10.1097/ACM.0000000000000388 24979285

[18] Marlowe DB, Theiss DS, Ostlie EM, Carnevale J. Drug court utilization of medications for opioid use disorder in high opioid mortality communities. J Subst Abuse Treat. 2022;1(141):108850. 10.1016/j.jsat.2022.108850 35931014

[19] Atlas.ti. Version 8. Berlin (GER): Atlas.ti Scientific Software Development GmbH. 2019. Available from: https://atlasti.com/.

[20] Guest G, Bunce A, Johnson L. How many interviews are enough?: An experiment with data saturation and variability. Field Methods. 2006;18(1):59–82.

[21] Hennink MM, Kaiser BN, Marconi VC. Code saturation versus meaning saturation: how many interviews are enough? Qual Health Res. 2017;27(4):591–608. 27670770 10.1177/1049732316665344 PMC9359070

[22] Averill JB. Matrix analysis as a complementary analytic strategy in qualitative inquiry. Qual Health Res. 2002;12(6):855–66. 12109729 10.1177/104973230201200611

[23] Adult drug court best practice standards. Alexandria (VA): National Association of Drug Court Professionals (US); 2024. Available from: https://allrise.org/wp-content/uploads/2024/05/Adult-Treatment-Court-Best-Practice-Standards-I-VI_VIII_X-final.pdf.

[24] Johns Hopkins Bloomberg School of Public Health, NYU Grossman School of Medicine, NaphCare, Yale University and National Harm Reduction Coalition, Rhode Island Department of Corrections, Viaduct Consulting LLC, et al. Medications for opioid use disorder in jails and prisons: moving toward universal access. Baltimore (MD): Johns Hopkins Bloomberg School of Public Health (US); 2021. Available from: https://americanhealth.jhu.edu/sites/default/files/2021-08/JHU-014%20OUD%20in%20Jail%20Report%20FINAL%202.pdf.

[25] National Academy for State Health Policy. States allowing telehealth prescriptions for opioid use disorder [Internet]. NASHP. 2021 [cited 2024 Jun 28]. Available from: https://nashp.org/state-tracker/states-allowing-telehealth-prescriptions-for-opioid-use-disorder/

[26] Mette E. Litigation prompting state and local correctional systems to change opioid use disorder treatment policies [Internet]. NASHP. 2021 [cited 2024 Jun 28]. Available from: https://nashp.org/litigation-prompting-state-and-local-correctional-systems-to-change-opioid-use-disorder-treatment-policies/

[27] Poulsen MN, Santoro W, Scotti R, Henderson C, Ruddy M, Colistra A. Implementation of telemedicine delivery of medications for opioid use disorder in Pennsylvania treatment programs during COVID-19. J Addict Med. 2023;17(2):e110–8. 36129690 10.1097/ADM.0000000000001079 PMC10022523

[28] Harrington C, Bailey A, Delorme E, Hano S, Evans E. “And then COVID hits”: a qualitative study of how jails adapted services to treat opioid use disorder during COVID-19. Subst Use Misuse. 2023;58(2):266–74. 36510800 10.1080/10826084.2022.2155480 PMC10208373

[29] Sufrin C, Kramer CT, Terplan M, Fiscella K, Olson S, Voegtline K, et al. Availability of medications for the treatment of opioid use disorder among pregnant and postpartum individuals in US jails. JAMA Netw Open. 2022;5(1):e2144369. 35050354 10.1001/jamanetworkopen.2021.44369 PMC8777564

[30] Booty MD, Harp K, Batty E, Knudsen HK, Staton M, Oser CB. Barriers and facilitators to the use of medication for opioid use disorder within the criminal justice system: perspectives from clinicians. J Subst Use Addict Treat. 2023;1(149):209051. 10.1016/j.josat.2023.209051 PMC10198928 37084815

[31] American Society of Addiction Medicine. Standards of care: for the addiction specialist physician. Chevy Chase: American Society of Addiction Medicine; 2014.

[32] Douds AS, Ahlin EM, Howard D, Stigerwalt S. Varieties of veterans’ courts: a statewide assessment of veterans’ treatment court components. Crim Justice Policy Rev. 2017;28(8):740–69.

[33] Ahmed FZ, Andraka-Christou B, Clark MH, Totaram R, Atkins DN, del Pozo B. Barriers to medications for opioid use disorder in the court system: provider availability, provider “trustworthiness”, and cost. Health Justice. 2022;10(1):24. 35895179 10.1186/s40352-022-00188-4 PMC9327334

[34] Pivovarova E, Evans EA, Stopka TJ, Santelices C, Ferguson WJ, Friedmann PD. Legislatively mandated implementation of medications for opioid use disorders in jails: a qualitative study of clinical, correctional, and jail administrator perspectives. Drug Alcohol Depend. 2022;1(234):109394. 10.1016/j.drugalcdep.2022.109394 PMC9169252 35349918

[35] Staton M, Pike E, Levi M, Lofwall M. The importance of justice and health care partnerships in MOUD feasibility trials. J Soc Work Pract Addict. 2023;0(0):1–15. 10.1080/1533256x.2023.2178119 PMC13459840 42583007

[36] Puglisi LB, Wang EA. Health care for people who are incarcerated. Nat Rev Dis Primer. 2021;7(1):1–2. 10.1038/s41572-021-00288-9 34238928

[37] Pont J, Enggist S, Stöver H, Williams B, Greifinger R, Wolff H. Prison health care governance: guaranteeing clinical independence. Am J Public Health. 2018;108(4):472–6. 29470125 10.2105/AJPH.2017.304248 PMC5844391

[38] Taylor EN, Timko C, Binswanger IA, Harris AHS, Stimmel M, Smelson D, et al. A national survey of barriers and facilitators to medications for opioid use disorder among legal-involved veterans in the Veterans Health Administration. Subst Abuse. 2022;43(1):556–63. 10.1080/08897077.2021.1975867 PMC9423124 34586978

[39] Scott CK, Dennis ML, Grella CE, Mischel AF, Carnevale J. The impact of the opioid crisis on U. S. state prison systems. Health Justice. 2021;9(1):17. 34304335 10.1186/s40352-021-00143-9 PMC8310396

[40] Brezel ER, Powell T, Fox AD. An ethical analysis of medication treatment for opioid use disorder (MOUD) for persons who are incarcerated. Subst Abuse. 2020;41(2):150–4. 10.1080/08897077.2019.1695706 31800376

[41] National Sheriffs’ Association. Jail-based medication-assisted treatment: promising practices, guidelines, and resources for the field. Alexandria (VA); National Sheriffs' Association (US); 2018. Available from: https://www.sheriffs.org/publications/Jail-Based-MAT-PPG.pdf.

[42] Substance Abuse and Mental Health Services Administration. Use of medication-assisted treatment for opioid use disorder in criminal justice settings. Rockville (MD); Substance Abuse and mental Health Services Administration (US); 2019. Available from: https://store.samhsa.gov/product/Use-of-Medication-Assisted-Treatment-for-Opioid-Use-Disorder-in-Criminal-Justice-Settings/PEP19-MATUSECJS.

[43] Weizman S, Perez J, Manoff I, Baney M, El-Sabawi T. A national snapshot: Access to medications for opioid use disorder in U.S. jails and prisons - litigation, legislation, and policies. Washington, D.C.; O’Neill Institute for National and Global Health Law (US); 2021. Available from: https://oneill.law.georgetown.edu/publications/national-snapshot-access-to-medications-for-opioid-use-disorder-in-u-s-jails-and-prisons/.

[44] *Estelle v. Gamble *(1976) 429 US 97.

[45] Council on Criminal Justice. Medicaid and reentry: policy changes and considerations for improving public health and public safety. Washington, D.C.; Council on Criminal Justice (US); 2022. Available from: https://counciloncj.org/health-and-reentry-project-brief-issue-1/.

[46] Bureau of Justice Assistance. Managing substance withdrawal in jails: a legal brief. Washington, D.C.; Department of Justice (US); 2022. Report No.: NCJ 304066.

