# Supplementary material for: Influences of the criminal justice system on use of medications for opioid use disorder: a qualitative study
Source: BMC Glob Public Health. 2024 Sep 19;2:64. doi: 10.1186/s44263-024-00093-y (PMC11622969; doi:10.1186/s44263-024-00093-y)
Supplement: Supplementary file 2 — Additional file 2: Qualitative Interview Guide [file 44263_2024_93_MOESM2_ESM.docx]

**Interview Guide**

The purpose of our study is to understand the unique experiences of justice-involved veterans with opioid use disorder. We want to learn about factors that may explain changes in your access to medications for opioid use disorder, consisting of methadone, buprenorphine, and naltrexone. By talking with you, we hope to learn from your experiences and assist the VA in making decisions about getting medication assisted treatment for opioid use disorder for justice-involved veterans.

I have just a few reminders before we get started. Please feel free to ask questions as we go along. There are no right or wrong answers. If you feel uncomfortable with any questions or need to take a break, please let me know. What we talk about today will be kept confidential. We'd like to audio record the interview to make sure we keep good track of our discussion today.

**We’d like to ask a few questions about you so that we can summarize information about the people we interviewed, *before* we turn on the recording for your privacy.**

1. Current position?

2. Gender?

3. Age?

4. Race?

5. Ethnicity?

6. Highest educational degree?

7. If we were to need to contact you again, is this your best email address and phone number?

Do you have any questions before we get started?

Is it ok with you if I turn on the audio recorder?

Consent: Do I have your consent to conduct this interview and audio record your answers?

**Background**

1. To get started, I’d like to learn a little bit about you. Where do you get your health care? Please tell me about your experiences getting health care.

**We understand that this health care system can be particularly challenging to work with. Can you relate to that statement and if so, share about a recent experience that has been difficult for you in getting treatment for opioid use disorder?**

1. Are you eligible for VA health care? Please tell me about your experiences at VA.

**Some participants have mentioned their courts or jails don’t provide or allow medications for OUD. Is that an experience that you have had and how has that affected you and your recovery?**

**Justice System Involvement**

1. Please tell me about your experiences with the law: jails, courts, prisons, etc. When was the first time you were involved with the justice system, which includes the police, court system, or jail/prison?
2. When was the most recent time you were involved with the justice system?
3. About how many times total have you had contact with the justice system? E.g., number of arrests, court trials, jail/prison sentences?
4. Are you comfortable talking about the reasons you were arrested or were involved with the justice system? Can you share those reasons?
5. Do you have any mental health issues or alcohol or drug use problems that have influenced you getting involved with the justice system?
6. Is your opioid use related to your contact with the justice system?

**Intervention Characteristics**

**Relative Advantage**

1. How much do you know about medications that are prescribed for opioid use disorder (e.g., methadone, buprenorphine, Suboxone, Zubsolv, Buprenex, Butrans, Probuphine, and Belbuca and naltrexone Revia and Vivitrol)?
   1. Have you ever taken any of these medications? Where did you get these medications?
2. What treatments work well for you?
   1. Do some treatments for your opioid use disorder work better than others? Why or why not?

**Evidence Strength & Quality**

1. Do any doctors or health care professionals you see tend to prefer or recommend certain treatments to you? What were their reasons for recommending certain treatments?
2. Are you familiar with the scientific evidence for pharmacotherapy for opioid use disorder? Would it matter to you to know the scientific evidence?

**Complexity**

1. Please walk me through a typical visit you have at VA. Is it different if you see a primary care provider, mental health provider, or addiction treatment provider? How so?
   1. How are treatment options discussed?
   2. Has a doctor discussed medication for opioid use disorder (methadone, buprenorphine, or naltrexone) as a treatment option with you?
2. Please walk me through a typical visit at a community health care provider. Who do you see? How is your visit different than at VA?
   1. How are treatment options discussed?
3. Has a doctor discussed medication for opioid use disorder (methadone, buprenorphine, or naltrexone) as a treatment option with you?

**Design Quality & Packaging**

1. When discussing treatment options, has a provider ever given you any resources? Online resources, marketing materials, or a toolkit?
2. What additional materials would be helpful for you to learn more about medications for opioid use disorder?

High performers: At your facility, it appears that XX% of veterans involved with the law used medication for opioid use disorders, compared to XX% for veterans not involved with the law.

- Are there any reasons you can offer as to why more veterans involved with the law at your facility might have higher rates of using medications for OUD?

Low performers: At your facility, it appears that XX% of veterans involved with the law used medication for opioid use disorders, compared to XX% for veterans not involved with the law.

- Do you think there are things getting in the way of using medications for OUD at your facility?

Increasing performers: At your facility, it appears that XX% of veterans involved with the law used medication for opioid use disorders in FY16 and increased to XX% in FY17.

- Are there any reasons you can offer as to why more veterans involved with the law at your facility might have used more medication assisted treatment for OUD over the last 2 years?

Decreasing performers: At your facility, it appears that XX% of veterans involved with the law used medication for opioid use disorders in FY16 and this number decreased to XX% in FY17.

- Do you think there are things getting in the way of of using medications for OUD at your facility that explains this decrease?

**Sometimes numbers don’t clearly reflect the quality of care that’s being provided, what do you think of these numbers? Would you say they accurately depict your experiences at your facility? Why/Why not?**

Outer Setting

**Patient Needs & Resources**

1. Do you prefer certain treatments for opioid use disorder?
2. Do medications for opioid use disorder help meet your needs? In what ways? How do they not meet your needs?
   1. What gets in the way with medications for opioid use disorder?

Characteristics of Individuals

**Knowledge & Beliefs about the Intervention**

1. What do you believe is the best treatment for opioid use disorder (e.g., detox options, inpatient treatment, outpatient psychosocial counseling, medications, combination)?
2. Before this interview, had you heard of any of the following medications for opioid use disorder: methadone, buprenorphine, or naltrexone (oral or injectable)?
3. Do you have any concerns related to medications for opioid use disorder? Why or why not?
4. Would you consider taking methadone, buprenorphine, or naltrexone (oral or injectable)?

**Other Personal Attributes**

1. What is your philosophy toward the treatment of substance use disorders in general (e.g., abstinence-orientation, harm reduction)?
   1. Toward treatment of opioid use disorder?

**Implementation Strategies**

1. You identified [barrier] earlier in the interview. One strategy to overcome such a barrier is [strategy]. Do you think such a strategy would be helpful for you? Why or why not?
2. You identified [facilitator] earlier in the interview. This is like [strategy]. Do you think this strategy would work for other justice-involved veterans? Why or why not?
3. Do you have any other ideas of ways that may help to ease the use of medications for opioid use disorder?
4. Do you think providing longer treatment would help you remain sober?
5. Would it be helpful to have academic information provided online?
6. Would you find it helpful to have a list of providers you can call for treatment?
7. How would you suggest dealing with the stigma surrounding medication assisted treatment for opioid use disorder?
8. Would you find it helpful to tailor treatment more specifically for justice involved veterans? How so?

**At the beginning of our interview you mentioned feeling challenged by […], which seems to be a common issue among other veterans as well. Is there anything you feel would help improve that situation?**

1. Before we finish, I’d like to ask if I missed anything I should know about your thinking about medication for OUD, or about the best ways to treat opioid dependence generally?
