# Supplementary material for: Influences of the criminal justice system on use of medications for opioid use disorder: a qualitative study
Source: BMC Glob Public Health. 2024 Sep 19;2:64. doi: 10.1186/s44263-024-00093-y (PMC11622969; doi:10.1186/s44263-024-00093-y)
Supplement: Supplementary file 3 — Additional file 3: Analysis Code Book [file 44263_2024_93_MOESM3_ESM.docx]

| **Qualitative Interview Code List** |
| --- |
| Barriers to MOUD Treatment |
| Facilitators to MOUD Treatment |
| Benefits of MOUD |
| Care Coordination Pathways with the Criminal Justice System |
| Patient Needs, Preferences, and Experiences |
| Provider and Criminal Justice Staff Experience and Beliefs about MOUD Treatment |
| Methods for Accessing Information about MOUD |
| Openness to Change MOUD Practices |
| Substance Use Disorder Treatment Philosophy |
| Veteran Culture |
| OUD Treatment for Non-VA Eligible Veterans |
| Care Coordination Between the VA & Community |
| OUD Treatment Types and Experiences |
| Treatment Policies in the Criminal Justice System |
| MOUD Implementation Strategies |
